# CARTA AO EDITOR

**DOI:** 10.1590/0034-7167.2022750603c

**Published:** 2022-07-08

**Authors:** Marcelo Prado Amaral-Rosa, Ana Cristina Pretto Báo

**Affiliations:** I Pontifícia Universidade Católica do Rio Grande do Sul, Rio Grande do Sul, Brasil.; II Universidade Federal do Rio Grande do Sul. Porto Alegre, Rio Grande do Sul, Brasil.; III Hospital de Clínicas de Porto Alegre. Porto Alegre, Rio Grande do Sul, Brasil.

CRÍTICA À ABORDAGEM DE PESQUISA E USO DO IRAMUTEQ

Ilma. Sra.

Profa. Dra. Dulce Aparecida Barbosa

Editora-Chefe Revista Brasileira de Enfermagem,

Por interesse em pesquisas que apresentam tratamento de dados com o software qualitativo IRaMuTeQ e abordagem metodológica mista, analisou-se o artigo *Segurança do paciente em situação de emergência: percepções da equipe de enfermagem*^(1)^. A considerar a abordagem metodológica e o uso do software IRaMuTeQ, aponta-se os seguintes aspectos:

I. o artigo não demonstra a combinação de técnicas/ tratamento de dados de forma complementar (sequencial e/ou convergente) no que se refere as abordagens quantitativa e qualitativa^(2)^, justificando a abordagem mista.
II. sobre a técnica de grupo focal, não há menção ao período de duração.
III. o uso *per si* do IRaMuTeQ não configura um método analítico^(3)^.
IV. a representação que organiza determinados fatores e variáveis é *dendrograma.*
V. “[...] constatou-se a ocorrência de 1.381 palavras, [...]. Isso se configurou em um critério empregado como ponto de corte para a inclusão dos elementos no dendograma (sic) [...]”, não é possível saber se o n. de ocorrência apresentado é o n. total de ocorrências e nem tampouco entender o procedimento realizado no IRaMuTeQ e o motivo do corte ser o dobro da frequência média.
VI. “Com embasamento na Classificação Hierárquica Descendente, foram analisados 38 segmentos de texto, em que 78,95% do corpus foi classificado para a elaboração das sete classes provenientes das partições de conteúdo (Quadro 1)”, trecho que coloca em suspeição todo o trabalho, uma vez que: vi.i) *corpus* pequeno para análise no IRaMuTeQ; ii) 38 STs é um n. baixíssimo; iii) índice de retenção suspeito (78,95%); iv) a CHD não é apresentada, o que causa profunda estranheza; vi) n. de classes (n=7) suspeito para o n. de STs^(4)^.
VII. no Quadro 1, não se sabe o que são e nem como surgiram as *partições*. Além disso, o somatório geral das porcetagens ultrapassa os 100% (Σ é 103,3%).
VIII. apresentados os excertos (sem score) de apenas três (T1, T5 e E2) dos sete participantes, sendo citado o mesmo participantes em diferentes *partições.*

Por fim, percebe-se diversas situações nebulosas, principalmente, frente ao uso do IRaMuTeQ, o que exige explicações por parte dos autores responsáveis pelo manuscrito.

## References

[1] Lima Gomes AT, Ferreira MA, Salvador PTCO, Bezerril MS, Chiavone FBT, Santos VEP (2019). Safety of the patient in an emergency situation: perceptions of
the nursing team. Rev Bras Enferm.

[2] Creswell JW (2013). Plano Clark VL. Pesquisa de métodos mistos.

[3] Martins ICS, Lima VMR, Amaral-Rosa MP, Moreira L, Ramos MG, Costa A, Reis L, Moreira A Computer Supported Qualitative Research. WCQR 2019. Advances in
Intelligent Systems and Computing.

[4] Ratinaud P (2009). IRAMUTEQ: Interface de R pour les analyses multidimensionnelles de
textes et de questionnaires [Computer Software][Internet].

