# Supplementary material for: CARTA AO EDITOR
Source: Rev Bras Enferm. 2022 Jul 8;75(6):e2022750603c. [Article in Portuguese] doi: 10.1590/0034-7167.2022750603c (PMC9728870; doi:10.1590/0034-7167.2022750603c)
Supplement: Related texts

# Reply: RESPOSTA DOS AUTORES

**Authors:** Viviane Euzébia Pereira Santos, Andréa Tayse de Lima Gomes

**Affiliations:** I Universidade Federal do Rio Grande do Norte. Natal, Rio Grande do Norte, Brasil.

 Prezada Professora Doutora Dulce Aparecida Barbosa Editora-chefe da Revista Brasileira de Enfermagem 

Inicialmente, destacamos que nós, autores do artigo intitulado *“Segurança do paciente em situação de emergência: percepções da equipe de enfermagem”*, prestamos estes esclarecimentos com estranheza e perplexidade, tendo em vista que o referido artigo foi avaliado pelos renomados e conceituados pareceristas que compõem o corpo de avaliadores *ad hoc* da Revista Brasileira de Enfermagem (REBEn), bem como passou pela aprovação do editor da REBEn.

Adiciona-se a isso que devido às argumentações vazias e sem fundamentação teórica robusta dos autores da carta enviada ao editor, supomos que não há *expertise* para expor os graves julgamentos quanto a qualidade e a validade dos resultados descritos no artigo. Apesar de haver o e-mail dos autores explícitos no artigo publicado, não recebemos nenhum contato para esclarecimentos sobre eventuais dúvidas relacionadas a publicação. Portanto, colocamo-nos a disposição para auxiliá-los no que for necessário e estiver ao nosso alcance quanto a questionamentos atrelados a pesquisa desenvolvida e publicada na REBEn.

Além disso, o referido artigo se trata de uma publicação do ano de 2019, cujo tratamento e análise de dados foram realizados em 2015. Desde então, tanto a ciência quanto os *softwares* voltados à pesquisa passaram por um processo evolutivo no que concerne ao conhecimento e a aplicabilidade em pesquisas^(1)^. Compreendemos e ressaltamos que os conhecimentos metodológicos se aperfeiçoam com o tempo e, portanto, é importante situar a produção científica na época em que foi produzida.

Posto isso, o *software Iramuteq* foi disponibilizado em língua francesa no ano de 2009^(2)^. Apenas em 2011 começou a ser adaptado para o português^(3)^ e desde a sua criação o tutorial passou por atualizações expressivas, inclusive com mudanças importantes quanto às normalizações de preparo do *corpus* textual.

Quanto aos aspectos explicitados na carta ao editor, reitera-se que:

I. No artigo, é apresentado que: *“[...] esse estudo faz parte de uma pesquisa maior [...]”*, por isso foi delineado como abordagem mista. No entanto, no recorte apresentado, reconhece-se atualmente que se trata de uma abordagem qualitativa, ainda que utilizadas as estatísticas textuais nos resultados.
II. O grupo focal teve duração de 4 horas e envolveu a apresentação do projeto de pesquisa e assinatura dos termos (consentimento livre e esclarecido, uso de imagens e gravação de voz); preenchimento do questionário de caracterização sociodemográfica e profissional dos participantes; apresentação da proposta e construção dos cenários; dissertação sobre o que foi construído nos cenários; discussão e compartilhamento dos resultados. Ressaltamos que em momento posterior todas as transcrições foram validadas pelos participantes.
III. Os dados não foram analisados pelo *software*. Ratifica-se que os dados foram tratados com suporte do *Iramuteq* e analisados quanto os aspectos lexicográficos pelos pesquisadores, conforme descrito no método do artigo: *“Para tanto, foram utilizadas a Classificação Hierárquica Descendente e a análise de similitude **como métodos de tratamento dos dados**”*. Os autores do manuscrito compreendem e difundem a concepção de que *softwares* constituem mecanismos de apoio à análise de dados. Destarte, devem ter seu uso fundamentado e consciente, com o papel central fundamental do pesquisador.
IV. Não se apresentou o dendrograma porque o desejável é que se apresente a análise dos resultados, e não um mero “copiar e colar” das figuras geradas pelos *softwares*. Enfatiza-se que não é regra a apresentação exata dos *outputs* do *software*, desde que os elementos centrais para interpretação dos dados sejam explicitados. No entanto, apresentamos abaixo a figura do dendrograma para fins de clareza quanto à análise.
V. O tutorial do *Iramuteq* esclarece sobre a importância quanto ao estabelecimento de pontos de corte correspondente a representações significativas das palavras, ou seja, a inclusão não deve ser embasada apenas na frequência em que as palavras apareceram, mas no ponto de corte definido^(3-4)^. Nesse caso, busca-se uma fundamentação teórica de expressividade dos vocábulos no *corpus* textual e, para tanto, o quociente entre o número de ocorrências e o número de formas é utilizado pelos pesquisadores, aspecto que foi abordado e discutido pelo próprio Pierre Ratinaud, criador do *software*, em 2020, ocasião em que esteve presente na Universidade Federal do Rio Grande do Norte.
VI. Ressalta-se que foi atingido o ponto de corte de aproveitamento, que é de no mínimo 75,0%^(4)^. E, para além do aspecto quantitativo de composição dos *corpus* textuais, o imprescindível é um preparo adequado do texto e a densidade teórica do *corpus* textual. Portanto, não é adequado que se conteste a validade de um corpus textual pelo simples fato de conter apenas 38 segmentos de texto, pois os resultados alcançados vão muito além do quantitativo de segmentos de texto analisados.
VII. Descrevemos as partições em forma de quadro para facilitar a compreensão do leitor sobre os principais resultados, o que não causa comprometimento à qualidade do estudo. Enviamos o dendrograma tal qual como gerado pelo *software* para que haja clareza quanto aos percentuais em cada classe (Figura 1).
VIII. Para a apresentação dos resultados, foram selecionadas representações correspondentes a cada classe.

**Figura 1:** Dendrograma gerado pelo *software Iramuteq*

Considerando que um dos benefícios do *software* é a transparência da análise dos dados, de maneira que pode ser recuperada em qualquer tempo, caso julgue necessário, podemos esclarecê-la quanto às demais representações componentes de cada classe.

Por fim, nos colocamos à disposição – Profa. Dra. Viviane Euzébia (e-mail: vivianeepsantos@gmail.com) e Andréa Gomes (e-mail: andrea.tlgomes@gmail.com) – para discutirmos e detalharmos como se procedeu o desenvolvimento da pesquisa, caso julgue necessário, inclusive demonstrar os resultados na íntegra do tratamento dos dados gerados pelo *software Iramuteq* e como se deu a análise com embasamento nos referenciais teóricos pertinentes.

## References

[1] Sousa YSO (2021). O Uso do Software Iramuteq: fundamentos de lexicometria para
pesquisas qualitativas. Estud Pesqui Psicol.

[2] Marchand P (2013). Quelques traces chronologiques de l'exploration
textométrique. Bulletin de Méthodologie Sociologique.

[3] Camargo BV, Justo AM (2013). IRAMUTEQ: um software gratuito para análise de dados
textuais. Temas Psicol.

[4] Camargo BV, Justo AM (2018). Tutorial para uso do software IRaMuTeQ: Interface de R pour les
Analyses Multidimensionnelles de Textes et de Questionnaires.
